# Growth, Antigenicity, and Immunogenicity of SARS-CoV-2 Spike Variants Revealed by a Live rVSV-SARS-CoV-2 Virus

**DOI:** 10.3389/fmed.2021.793437

**Published:** 2022-01-07

**Authors:** Limin S. Ding, Yuhang Zhang, Dan Wen, Jianbo Ma, Hao Yuan, Hongyue Li, Shuguang Duo, Fei Yuan, Yong E. Zhang, Aihua Zheng

**Affiliations:** ^1^State Key Laboratory of Integrated Management of Pest Insects and Rodents, Institute of Zoology, Chinese Academy of Sciences, Beijing, China; ^2^CAS Center for Excellence in Biotic Interactions, University of Chinese Academy of Sciences, Beijing, China; ^3^State Key Laboratory of Stem Cell and Reproductive Biology, Institute of Zoology, Chinese Academy of Sciences, Beijing, China; ^4^Key Laboratory of Zoological Systematics and Evolution, Institute of Zoology, Chinese Academy of Sciences, Beijing, China; ^5^Laboratory Animal Center, Institute of Zoology, Chinese Academy of Sciences, Beijing, China; ^6^CAS Center for Excellence in Animal Evolution and Genetics, Chinese Academy of Sciences, Kunming, China; ^7^Key Laboratory of Tropical Translational Medicine of Ministry of Education, School of Tropical Medicine and Laboratory Medicine, Hainan Medical University, Haikou, China

**Keywords:** SARS-CoV-2, spike, mutation, antigenicity, immunogenicity, infectivity, variant, VSV

## Abstract

SARS-CoV-2 is an emerging coronavirus threatening human health and the economy worldwide. As an RNA virus, variants emerge during the pandemic and potentially influence the efficacy of the anti-viral drugs and vaccines. Eight spike variants harboring highly recurrent mutations were selected and introduced into a replication-competent recombinant VSV in place of the original G protein (rVSV-SARS-CoV-2). The resulting mutant viruses displayed similar growth curves *in vitro* as the wild-type virus and could be neutralized by sera from convalescent COVID-19 patients. Several variants, especially Beta strain, showed resistance to human neutralizing monoclonal antibodies targeting the receptor-binding domain (RBD). A single dose of rVSV-SARS-CoV-2 Beta variant could elicit enhanced and broad-spectrum neutralizing antibody responses in human ACE2 knock-in mice and golden Syrian hamsters, while other mutants generated antibody levels comparable to the wild-type. Therefore, our results will be of value to the development of next-generation vaccines and therapeutic antibodies.

## Introduction

COVID-19 is a highly transmissible and life-threatening disease that emerged in December 2019. As of September 2021, more than 223 million confirmed cases and 4.6 million deaths were reported worldwide by WHO. The etiological agent of COVID-19, SARS-CoV-2, causes symptoms ranging from mild to lethal pneumonia and even multiple organ failure (1, 2).

SARS-CoV-2 is single-strand positive RNA virus belonging to the family *Coronaviridae*, genus *Betacoronavirus*. The genome of SARS-CoV-2 encodes four structural proteins, including spike (S), envelope (E), matrix (M), and nucleocapsid (N) (3). S protein is the major glycoprotein on the surface of SARS-CoV-2 and is responsible for receptor binding and membrane fusion (4, 5). S protein is processed by the host's furin protease into S1 and S2 (6). S1 interacts with receptor ACE2 through the receptor-binding domain (RBD), which is also the main target of the neutralizing antibodies (7). S2 mediates fusion between the viral envelop and the host cell membrane (6). Vaccines and therapeutic antibodies are key tools to control the pandemic of COVID-19 as vaccines developed by various platforms, such as mRNA, inactivated virus, adenovirus, and subunit vaccines, were approved for emergency use (8, 9). In addition, many therapeutic antibodies for COVID-19 are tested in clinical trials (10, 11).

RNA viruses have a higher mutation rate than DNA viruses (12). Amino acid changes in the viral surface proteins can significantly affect antigenicity, pathogenicity, and transmission. For example, a single serine-to-asparagine substitution (S139N) in the prM protein of the Zika virus contributes to fetal microcephaly (13). An A226V mutation in the E1 protein of the Chikungunya virus (CHIKV) enhanced the vector competence of *Aedes albopictus*, largely driving the fast spreading of CHIKV from Africa to Asia and the West hemisphere (14).

Since D614G became the dominant strain of SARS-CoV-2, the major emerging variants were divided into two categories, one was classified as “variants of concern” including alpha, beta, gamma and delta variants, the other was “variants of interest” like epsilon, eta, lota and kappa variants. Among them, the alpha and beta variants were the most studied variants. Alpha variant from UK was illustrated to be sensitive to neutralization by most RBD-directed monoclonal antibodies (mAbs) and similarly neutralization to patient sera recovered from COVID-19 compared with D614G (15, 16). However, beta variant from South Africa was demonstrated to be more resistant to mAbs and plasma from convalescent patient and vaccinated individuals (16–18). As for cross-variant neutralization, gamma variant from Brazil and beta variant were regarded tough to neutralize on account of evading antibody responses induced upon infection and vaccination, as well as therapeutic antibodies compared to alpha (19, 20), while gamma variant was revealed less resistance to beta in the meantime (20). In addition, delta variant from India was also identified resistant to antibodies raised by infection and vaccination (21).

This study investigated the biological significance of natural circulating variants with mutations in the S protein of SARS-CoV-2. To achieve this, we generated the replication-competent recombinant VSVs expressing eight S variants (22). Besides the infectivity and reactivity to neutralizing antibodies, we also determined the immunogenicity of the variants in a hACE2 knock-in mouse model.

## Materials and Methods

### Ethics Statement

All rVSV recombinant virus system studies were performed under biosafety level 2 (BSL2) conditions. All mice experiments were conducted strictly according to the bioethics guidelines and were approved by the Bioethics Committee of the Institute of Zoology, Chinese Academy of Sciences (Approval number: IOZ-IACUC-2020-036). Human sera samples of COVID-19 convalescent patients were collected from the Department of Virology, State Key Laboratory of Pathogen and Biosecurity, Institute of Microbiology and Epidemiology, Academy of Military Medical Sciences, Beijing, China. Written informed consents were provided by the patients before the study. All sera samples were incubated at 56°C for 30 min to inactivate the complement before experiments (22).

### Plasmids

rVSV vector plasmid was designed, synthesized, and constructed as described previously (23). Briefly, firefly luciferase encoding sequences were added at nt72 to generate the rVSV-Luc-G plasmid for use as the backbone for other plasmids. S from strain Wuhan-Hu-1 (GenBank: MN908947) was then codon-optimized for human cells and cloned into rVSV-Luc-G plasmid, replacing the VSV glycoprotein coding sequence (3845–5380). The resulting plasmid rVSV-Luc-SARS-CoV-2 (rVSV-S^WT^) (wildtype) was used as the template and modified to different variants by site-directed mutagenesis. Primers for mutations were V367F: sense 5′-gtggccgactactccttcctgtacaactccgcc-3′; antisense 5′-ggcggagttgtacaggaaggagtagtcggccac-3′; G476S: sense 5′-gagatttaccaggccagctctaccccatgcaac-3′; antisense 5′-gttgcatggggtagagctggcctggtaaatctc-3′; V483A: sense 5′-cccatgcaacggcgcagagggcttcaact-3′; antisense 5′-agttgaagccctctgcgccgttgcatggg-3′; D614G: sense 5′-ccgtgctgtaccagggagtgaactgcaccga-3′; antisense 5′-tcggtgcagttcactccctggtacagcacgg-3′; D839Y: sense 5′-attaagcagtacggctactgcctgggcgacatt-3′; antisense 5′-aatgtcgcccaggcagtagccgtactgcttaat-3′; D936Y: sense 5′-attggcaagattcagtactctctgagcagcaca-3′; antisense 5′-tgtgctgctcagagagtactgaatcttgccaat-3′. Alpha variant: N501Y mutation, sense 5′-cttccagcctacatacggcgtgggctaccagc-3′, antisense 5′-gctggtagcccacgccgtatgtaggctggaag-3′; D614G mutation, sense 5′-ccgtgctgtaccagggagtgaactgcaccga-3′, antisense 5′-tcggtgcagttcactccctggtacagcacgg-3′. Beta variant: K417N mutation, sense 5′-gccagacaggcaatatcgccgactac-3′, antisense 5′-gtagtcggcgatattgcctgtctggc-3′; E484K mutation, sense 5′-gcaacggcgtgaagggcttcaactg-3′; antisense 5′-cagttgaagcccttcacgccgttgc-3′; N501Y mutation, sense 5′-cttccagcctacatacggcgtgggctaccagc-3′, antisense 5′-gctggtagcccacgccgtatgtaggctggaag-3′; D614G mutation, sense 5′-ccgtgctgtaccagggagtgaactgcaccga-3′, antisense 5′-tcggtgcagttcactccctggtacagcacgg-3′.

### Cells, Antibodies, and Proteins

HEK293T cells (American Type Culture Collection [ATCC], CRL-3216), and Vero cells (ATCC, CCL-81) were maintained in Dulbecco's modified Eagle's medium (DMEM) supplemented with 8% fetal bovine serum (FBS), 1% L-glutamine, and 1% Penicillin-Streptomycin solution (P/S) at 37°C with 5% CO_2_.

MAbs of SARS-CoV-2 were gifts from Dr. Linqi Zhang's lab at Tsinghua University (24). These mAbs were screened from B cells of convalescent patients of COVID-19 using SARS-CoV-2 RBD as bait and showed no obvious overlapping epitopes with each other as confirmed by competitive ELISA. Rabbit anti-RBD polyclonal antibody against SARS-CoV-2 S protein (Cat. 40592-T62, Sino Biological, China) was purchased from Sino Biological Inc (Beijing, China). Dr. Xinquan Wang's lab generously provided ACE2 protein at Tsinghua University (25).

The sera were obtained during Feb, 2020 from COVID-19 convalescent patients all infected by Wuhan-Hu-1 strain (SARS-CoV-2 WT) (22). The ages of patients ranged from 21 to 57, among which three were severe, three were moderate and two were mild.

### Genome Analysis

SARS-CoV-2 genome and S protein sequences were downloaded from the Global Initiative for Sharing All Influenza Data (GISAID) on November 5, 2020, which used for constructing a phylogenetic tree and identifying mutations. On the GISAID website (https://www.gisaid.org/), download settings “complete” and “high coverage” were checked (26, 27). For the “Host,” “Human” was selected on the drop-down menu, and 131,448 sequences were downloaded in total.

The S protein sequences were back-translated to the S gene sequences on the SARS-CoV-2 genome (28). A sequence screening process was conducted based on the following criteria:

1. The number of “N” in the whole genome is smaller than 21;
2. The number of “N” in the back-translated S gene is smaller than 4;
3. The number of “X” in the back-translated S gene is smaller than 4;
4. The length of the back-translated S gene is less than or equal to 5,000, and the start position on the SARS-CoV-2 genome is greater than or equal to 19,000.

A multiple sequence alignment of 4,207 non-redundant SARS-CoV-2 S gene sequences as of May 2020 was performed using MAFFT (29). The alignment result was used to construct a phylogenetic tree by IQ-TREE (30). The S protein sequences were likewise aligned using MAFFT. The result was parsed by custom python code to provide the corresponding protein mutation information. Finally, the phylogenetic tree with annotated mutation information was visualized using the ggtree package in R (31–33).

Sequences of SARS-CoV-2 carrying corresponding mutations, which used for plotting histograms of monthly frequencies were different from those mentioned above. A new batch of SARS-CoV-2 genome and S protein sequences were downloaded from GISAID on January 20, 2021. On the GISAID website, download settings “complete,” “collection date compl,” and “high coverage” were checked. For the “Host,” “Human” was selected on the drop-down menu, and 265,195 sequences were downloaded.

The back-translation, screening, S protein alignment, and mutation calling process were the same as described above. Bar charts were plotted using the ggplot2 package in R (34).

### Recombinant VSV

rVSVs were rescued by reverse genetics methods as described previously (22). Briefly, HEK293T cells in 6 cm dishes were transfected with rVSV backbone plasmid (1.6 mg) and five supporting plasmids encoding T7 polymerase (8.1 mg), N (1.286 mg), P (639 ng), M (169.9 ng), and L (169.9 ng) by calcium phosphate transfection. The recovered viruses were confirmed by cytopathic effects and subsequently passaged on Vero cells.

The rVSVs were tittered on Vero cells by focus-forming assays. Vero cells were seeded in 96-well plates with 2.0 × 10^4^ cells/well in triplicate. Viruses to be examined were infected with 10-fold serial dilution at 28°C and incubated with DMEM containing 2% FBS plus 20 mM NH_4_Cl after 3 h infection. The virus titers were determined by immunofluorescence assays using the anti-S polyclonal antibody at 24 h post-infection.

Plaques formed by rVSVs were measured on Vero cells. Cells were infected by rVSVs in 24-well plates with ten-fold dilution in triplicate. The cells were then incubated in DMEM containing 2% FBS and 2% CMC-Hanks at 28°C after 3 h infection. Viral plaques were generally formed within 7 days and stained with 1% crystal violet.

### Western Blot

Twenty-milliliter rVSV stocks with titers of about 10^6^ FFU/ml were ultra-centrifuged with a 25% sucrose cushion at 39,000 rpm (SW41 rotor, Beckman, Fullerton, CA, USA) 4°C for 3 h and pellets were resuspended in 100 μl Phosphate-buffered saline (PBS). rVSV-infected cells were lysed with RIPA lysis buffer in accordance with the manufacturer's manual (Cat.89901, Thermo Fisher Scientific, USA). Samples from supernatants and cell lysates were separated by 8% SDS-PAGE and immunoblotted with anti-RBD polyclonal antibody against SARS-CoV-2 S protein (Cat. 40592-T62, Sino Biological, China) and anti-GAPDH mAb (Cat KM9002, Sungene biotech, China), each at a 1:2,000 dilution.

### Focus-Forming Assays

rVSV-infected cells were fixed with 4% paraformaldehyde (PFA) at room temperature (RT) for 1 h at 24 h post-infection. After being permeabilized with 0.05% Triton X-100 in PBS for 10 min and washed three times, the cells were incubated with rabbit polyclonal antibody recognizing the S RBD of SARS-CoV-2 (1:1,000) in 3% bovine serum albumin in PBS at RT for 1 h. After three washes with PBS, cells were incubated with Alexa Fluor 488 conjugated goat anti-rabbit secondary antibody (A-11034, Thermo Fisher Scientific, USA; 1:500) at RT for 30 min. After staining the nuclei with Hoechst 33342 (1 mg/ml) for 10 min, the cells were examined by confocal microscopy (Zeiss LSM 710, Germany).

### Virus Growth Curve

Vero cells in T25 flasks were infected with rVSVs at a multiplicity of infection (MOI) of 0.01. Supernatants were collected at indicated time points post-infection and tittered by focus-forming assays on Vero cells.

### Luciferase Assays

Vero cells were seeded in 384-well plates at 6.6 × 10^3^ cells/well in triplicate 24 h before infection. After infection with rVSVs for 24 h, the culture medium was incubated with 30 ml luciferase substrate (Bright-Glo Luciferase Kit, Promega, USA). After incubation for 5 min at RT, 60 ml lysates were transferred to black flat bottom 384-well plates and scanned by a microplate luminometer (PerkinElmer, USA).

Neutralization of rVSVs by sera and mAbs was performed as described in (22). Briefly, Vero cells were seeded in 384-well plates at 6.6 × 10^3^ cells/well 24 h before infection. Human sera or mAbs were two-fold serially diluted in duplicate and incubated with 1,000 FFU rVSVs for 1 h at 37°C. Mixtures were then added to cells in 384-well plates and incubated at 37°C for 24 h with 5% CO_2_. The luminescence was measured as described above. IC50 values were calculated by the Reed-Muench method (35).

Neutralization of rVSVs against sera from immunized hamster by authentic virus was performed in certified BSL-3 laboratory. Vero cells were seeded in 96-well plates with 80% confluence at the time of infection. Sera from immunized hamster were two-fold serially diluted in duplicate and incubated with 100 TCID50/well authentic SARS-CoV-2 Wuhan-Hu-1 strain at 37°C for 1 h. The cytopathic effect (CPE) of each well were recorded on day 4 and evaluated under microscopes. Neutralization titers were correspondingly calculated as the highest dilution of sera revealing 50% inhibition activity of authentic virus (IC50).

### Animal Immunization

The human ACE2 (*hACE2*) knock-in mouse model was developed by the Institute of Zoology, Chinese Academy of Sciences, Beijing, China. Briefly, the specific sgRNA, Cas9 mRNA, and the donor vector containing homology arm and hACE2 cDNA with BGH PolyA were mixed and microinjected into the pronuclei of ICR zygotes. Then, the injected zygotes were transferred into the oviducts of pseudo-pregnant foster mother mice. Finally, the *hACE2* cDNA was inserted following the control sequence of mouse ACE2.

Six to eight-week-old female hACE2 mice were immunized with a single dose of rVSV via the intraperitoneal injection (i.p.) route (200 μl per mouse). Morbidity and weight were monitored for 7 days post-vaccination. In addition, serum samples were collected 28 days post-vaccination for evaluating NAb titers against rVSV-S^WT^ or rVSV-S variants.

Five golden Syrian hamster per group were immunized respectively with rVSV-S^WT^ and rVSV-S^Beta^ via intramuscular and intranasal injection routes (10^5^ PFU/hamster). Sera samples were collected 30 days post-vaccination and the neutralizing titers were evaluated by live SARS-CoV-2 neutralization assays.

### Statistical Analyses

Statistical analyses were performed by GraphPad Prism 9.0 software (GraphPad Software, San Diego, CA, USA.). Quantitative data in the paper were displayed in mean ± SD form. Statistical significance was identified by ANOVA analyses for multiple comparisons in GraphPad Prism 9.0. Differences with *p* < 0.05 were regarded as statistically significant. IC50 (EC50) values in the paper were all calculated by the Reed-Muench method.

## Results

### Construction of rVSV-SARS-CoV-2 Natural Variants

**Figure 1:**
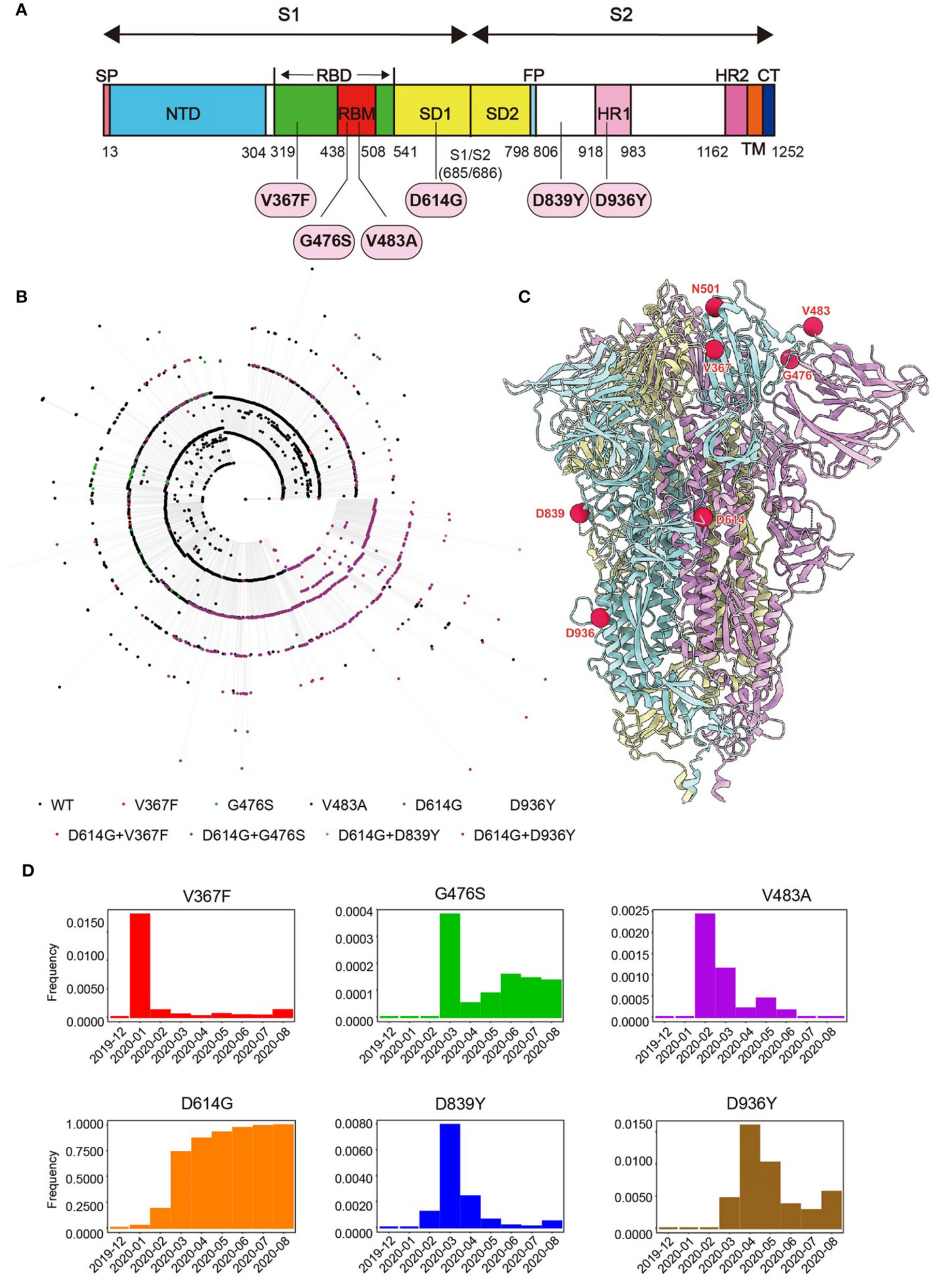
Genome analysis of SARS-CoV-2. **(A)** Schematic diagram of SARS-CoV-2 S protein. The mutations studied in the paper were marked. **(B)** Phylogenetic relationship between 4207 non-redundant SARS-CoV-2 S gene sequences as of May 2020. Different colors of external nodes represent sequences carrying the corresponding mutations. **(C)** The mutations studied in the paper were highlighted in the Cryo-EM structure of SARS-CoV-2 S trimer in pre-fusion conformation. Each S monomer is colored cyan, violet, or yellow. **(D)** Monthly frequencies of SARS-CoV-2 sequences carrying the corresponding mutations from December 2019 to August 2020 are demonstrated in the histogram.

To study the emerging variants of SARS-CoV-2 during the pandemic, we collected 24,743 SARS-CoV-2 sequences reported in the GISAID database on May 21, 2020 and compared them with the reference Wuhan-Hu-1 stain (MN_908947). Phylogenetic analysis was performed to pick mutations representing major subclades (Figure 1B). Six single-mutation variants that are highly recurrent (Frequency > 0.1%) and located in function-related domains were selected (Figures 1A,C). Among them, three mutations (V367F, G476S, and V483A) are in the RBD of the S1 subunit, which might affect the interaction between S and ACE2. The most dominant variant carries the D614G mutation, which is located outside the RBD of the S1 subunit. Both D839Y and D936Y are in the S2 subunit, and the latter is in the heptad repeat domain 1 (HR1) fusion core. In addition, from a structural perspective, D839Y might be involved in stabilizing the interaction with the TCR, while D936Y might affect the post-fusion state of S protein (36, 37). Notably, the frequency of these mutations peaked from January to March 2020 and then rapidly decreased except for D614G (Figure 1D; Supplementary Figure 1).

**Figure 2:**
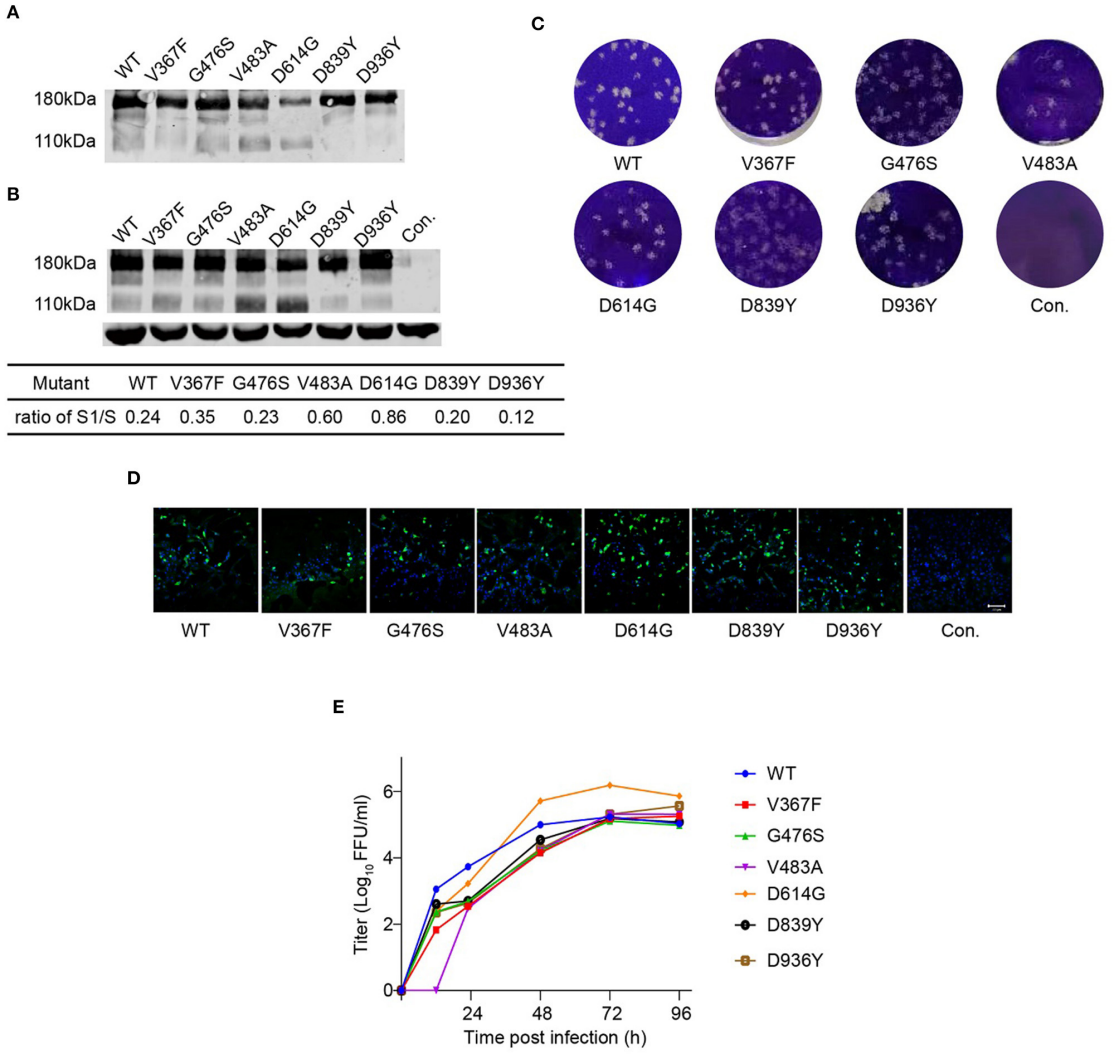
Characterization of rVSV-S variants. Purified rVSVs **(A)** and lysate of rVSV producing cells **(B)** were analyzed by western blot using a polyclonal antibody recognizing the RBD domain of the S protein with GAPDH as input control. The result is a representative of three independent experiments. The S1/S ratio was quantified by the gray values in Image Studio Ver 5.2 Software (LICOR Biosciences, USA) and significant difference was calculated by GraphPad Prism 9.0. **(C)** Plaque morphology of rVSV-S variants in Vero cells at 120 h post-infection. Control (Con.) is mock-infected. **(D)** Images of rVSV-infected Vero cells stained with serum from a convalescence patient (green). Nuclei were stained by Hoechst (blue). **(E)** The growth curve of rVSVs was tested in Vero cells at an MOI of 0.01. Viral titers were measured by a focus-forming assay and expressed as focus-forming units per ml (FFU/ml) (*n* = 3). Error bars indicate SD. The result is representative of three independent experiments. WT: blue; V367F: red; G476S: green; V483A: violet; D614G: orange; D839Y: black; D936Y: brown.

Previously, we reported a replication-competent recombinant VSV (rVSV) harboring the S protein of the SARS-CoV-2 Wuhan-Hu-1 strain, assigned as rVSV-SARS-CoV-2 (22). Here, we inserted a luciferase reporter into the rVSV-SARS-CoV-2, assigned as rVSV-Luc-SARS-CoV-2 (rVSV-S^WT^). Six rVSV variants (V367F, G476S, V483A, D614G, D839Y, and D936Y) were constructed using site-directed mutagenesis and rescued in 293T cells, and then passaged in Vero cells. The expression of SARS-CoV-2 S protein in purified virions and virus-infected cell lysates was tested by western blot showing a major 180 KD band and a minor 110 KD band, corresponding to full-length S and S1, respectively (Figures 2A,B). The expression level of S was comparable in the samples except for D614G, which showed a reduced full-length S protein. In addition, all the rVSVs formed plaques in Vero cells, with similar plaque size and morphology (Figure 2C). Meanwhile, mutated S proteins expressed in the infected cells could be recognized by serum from COVID-19 convalescence patients, suggesting that the S variants maintained a correct conformation (Figure 2D).

Vero cells were infected by rVSVs at an MOI of 0.01, and the growth curves of viruses in the supernatant were measured by a focus-forming assay (Figure 2E). All the rVSVs exhibited similar growth kinetics. They reached the maximum titers at 72 h post-infection and replicated at comparable levels. D614G variant displayed nine-fold higher viral titers than rVSV-S^WT^, suggesting that D614G mutation increased the replication efficiency of SARS-CoV-2. These data indicated that most of the mutants have no obvious effect on viral growth.

The infectivity of rVSV-S^WT^ and six variants was also determined by luciferase measurement with relative luminescence units (RLU) as the readouts. Again, there was a strong linear correlation between the luciferase activity and the log-transformed virus dilution, with R^2^ ranging from 0.92 to 0.99 (Supplementary Figure 2).

### Reactivity of Natural Variants to the Convalescent Sera

**Figure 3:**
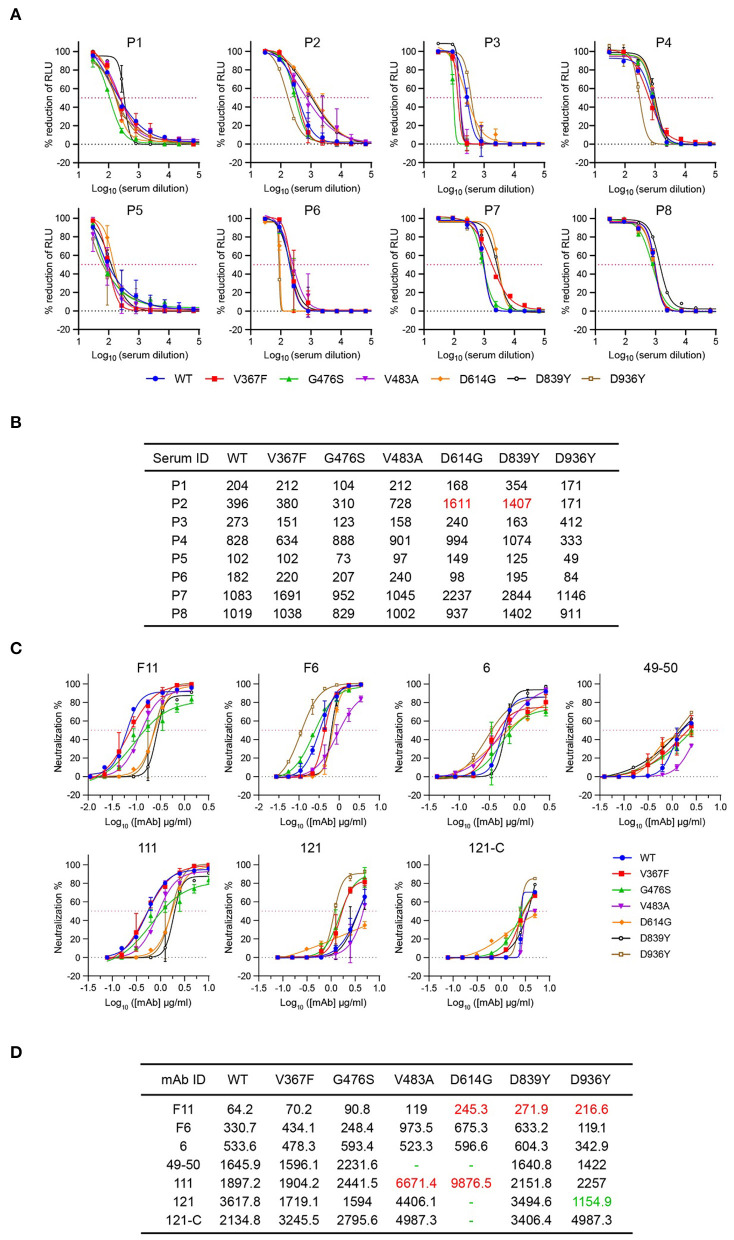
Neutralization activity of sera from COVID-19 convalescence patients and mAbs targeting RBD against rVSV-S variants. **(A)** Eight sera samples were tested against rVSV-S^WT^ and six variants using the focus reduction neutralization test. Error bars represent SD. All neutralization assays were performed in triplicate. **(B)** ID50 values of sera against rVSVs variants more than three-fold higher than those of WT are highlighted in red. **(C)** Seven mAbs were tested against rVSV-SWT and six variants using the focus reduction neutralization test (FRNT). Error bars represent SD. All neutralization assays were performed in triplicate. **(D)** IC50 values (ng/ml) of sera against rVSVs variants more than three-fold higher or lower than those of WT are highlighted in red or green, respectively. Dashes indicate values higher than 5,000.

The neutralizing activity in the convalescent serum samples strongly correlates with protective immunity (38). To determine whether sera from COVID-19 convalescent patients could neutralize circulating SARS-CoV-2 variants, we tested the neutralizing sensitivity of eight serum samples against rVSV-S^WT^ or variants. The neutralization titers of each serum were measured by a focus reduction neutralization test (FRNT). The IC50 of the eight sera tested ranged from 102 to 1083 against rVSV-S^WT^. None of the variants showed significant differences in neutralization sensitivity to all the eight sera, where a three-fold difference in neutralization titers compared with rVSV-S^WT^ was regarded as significant (Figures 3A,B; Supplementary Figure 3). The three variants carrying mutations in the RBD domain showed comparable neutralization sensitivity to all the human sera. Only human serum P2 exhibited higher neutralization titers against D614G and D839Y by 4.1- and 3.6-fold, respectively. In addition, the neutralization sensitivity of D936Y to four sera was slightly decreased by less than three-fold. Overall, the neutralization sensitivity of human convalescent sera against variants was similar to that against rVSV-S^WT^.

### Significant Differential Reactivity of Natural Variants to the Monoclonal Antibodies

MAbs targeting important epitopes of S protein are promising treatments for mild to moderate COVID-19. Therefore, we evaluated the susceptibility of these rVSVs to neutralization by seven mAbs targeting the S RBD domain of SARS-CoV-2 (22), where a three-fold difference in neutralization titers compared with rVSV-S^WT^ was regarded as significant. For neutralization of the three variants carrying RBD mutations, only the activities of 49–50 to G476S and 111 to V483A were impaired (Figures 3C,D). However, D839Y and D936Y became more resistant to neutralization by F11. Notably, D614G was more resistant to most of the antibodies except antibody 6 and F6. In contrast, mAb 121 exhibited a 3.3-fold lower neutralizing activity against D936Y, suggesting D936Y was more susceptible to neutralization by mAb 121 relative to the rVSV-S^WT^. These results demonstrated that the neutralization activities of mAbs are very sensitive to the emerging mutations in the S protein.

### Immunogenicity of S Variants

**Figure 4:**
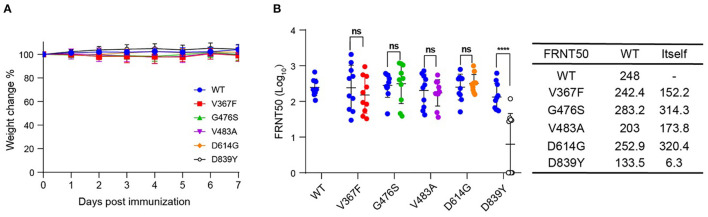
Immunogenicity of SARS-CoV-2 S variants. Groups of female hACE2 knock-in ICR mice (*n* = 10) were vaccinated with a single dose of rVSV-SWT or variants *via* i.p. route (3 × 10^5^ FFU/animal). **(A)** Following vaccination, weight changes were monitored for 7 days. **(B)** NAb titers against rVSV-SWT or the same variants were determined by FRNT and calculated by the Reed-Muench method at 30 days post-vaccination. Group geometric mean titers (GMTs) were indicated. Error bars indicate SD (*n* = 3). The result is representative of three independent experiments.

To further study the immunogenicity of the S protein variants, we immunized hACE2 knock-in ICR mice with a single shot of 3 ×10^5^ FFU corresponding recombinant viruses *via* the i.p. route. Mice weight was monitored for 7 days after immunization, and no obvious weight loss was observed (Figure 4A). None of the immunized mice showed clinical illness. Mice sera were collected at 30 days post-immunization, and the neutralization activity was analyzed by FRNT assay against the rVSV-S^WT^ and the homogeneous variant. The neutralization titers of V367F, V483A, D614G, and D839Y immunized serum samples against rVSV-S^WT^ were comparable to those against the corresponding homogeneous variants, with differences lower than two-fold (Figure 4B). Moreover, the serum neutralizing activity elicited by these four variants was as effective as that by rVSV-S^WT^. Notably, immunization of rVSV-S^D839Y^ stimulated a similar level of neutralizing antibodies against rVSV-S^WT^. However, the neutralization activity decreased eight-fold against the homogeneous strain. There was no significant difference between them except D839Y. Based on these results, rVSVs expressing the S variants could produce neutralizing antibodies in mouse models, with comparable titers to rVSV-S^WT^.

### Characterization of the Recently Identified Alpha and Beta Variants

**Figure 5:**
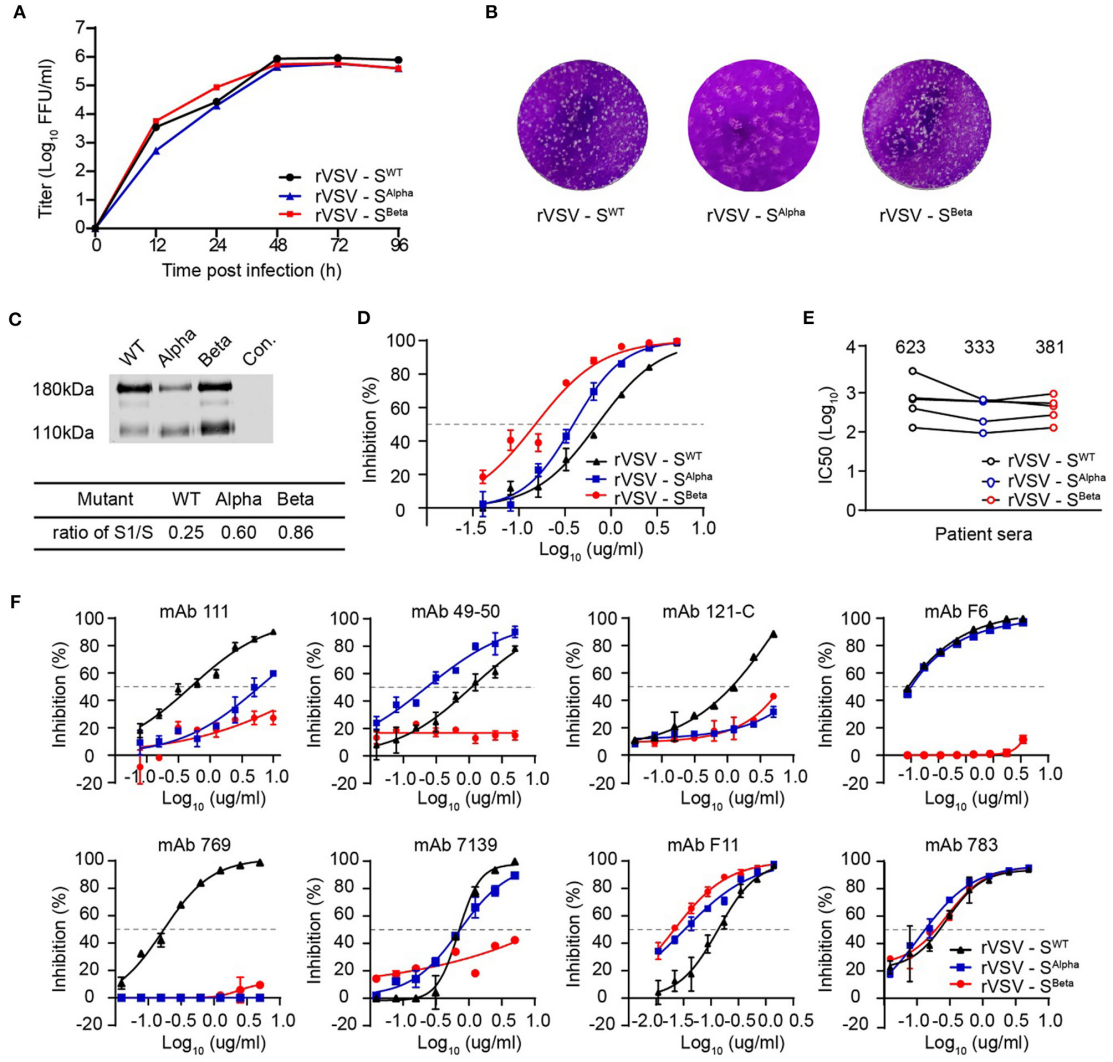
Characterizations of recently emerged variants of concern, Alpha and Beta. **(A,B)** The growth kinetics and plaque morphology of rVSV-S Alpha and Beta variants determined in Vero cells as above. The result is representative of three independent experiments. **(C)** Purified rVSVs were analyzed by western blot using a polyclonal antibody recognizing the RBD domain of the S protein. The result is representative of three independent experiments. The S1/S ratio was quantified by scanning gray values in Image Studio Ver 5.2 Software (LICOR Biosciences, USA) and calculated significant differences by GraphPad Prism 9.0. **(D)** The inhibitory activity of soluble ACE2 against rVSV-S^WT^, Alpha, and Beta variants. **(E)** Neutralization activity of sera from COVID-19 convalescence patients against rVSV-S variants. Using the FRNT, five sera samples were tested against rVSV-S^WT^, Alpha, and Beta variants. **(F)** Neutralization activity of mAbs against rVSV-S variants. The FRNT tested eight mAbs against rVSV-S^WT^, Alpha, and Beta variants. Error bars represent SD. All neutralization assays were performed in triplicate.

Since September 2020, a new SARS-CoV-2 variant B.1.1.7 (also known as 501Y.V1 or Alpha) emerged in southeast England and rapidly became the dominant strain in the UK. In the S gene of B.1.1.7, there are three deletions in the N-terminal domain (NTD) and six missense mutations in addition to D614G, including one mutation in the RBD domain (N501Y). In late 2020, the SARS-CoV-2 B.1.351 lineage (also known as 501Y.V2 or Beta) was first reported in South Africa and then spread to other countries. B.1.351 contains three substitutions in the RBD and a cluster of mutations in the other regions of the S gene in addition to D614. Although both B.1.1.7 and B.1.351 bear the N501Y mutation, they are phylogenetically different. To reveal the ability of B.1.1.7 and B.1.351 to induce neutralizing antibodies, we generated two recombinant VSVs expressing the SARS-CoV-2 S protein with D614G and their representative mutations in the RBD domain. Therefore, the S protein-carrying the double mutation (N501Y and D614G) was designated as S^Alpha^, whereas that with four mutations (K417N, E484K, N501Y, and D614G) was S^Beta^. Both rVSV-S^Alpha^ and rVSV-S^Beta^ were replication-competent in Vero cells, showing similar kinetics and plaque morphology as rVSV-S^WT^ (Figures 5A,B). rVSV-S^Alpha^ and rVSV-S^Beta^ showed a significantly increased S1/S ratio of 0.60 and 0.86 compared with the 0.25 of rVSV-S^WT^, respectively (Figure 5C). The receptor-binding affinities were gauged by measuring the IC50 of soluble ACE2 on rVSVs as above. Soluble ACE2 protein could neutralize rVSV-S^Alpha^ and rVSV-S^Beta^ infection, with IC50 values decreased by 1.8- and 4.7-fold compared with rVSV-S^WT^, respectively, implying stronger interaction between S^Beta^ and ACE2, followed by S^Alpha^ and then S^WT^ (Figure 5D).

Next, we determined the neutralization sensitivity of five human convalescent sera against rVSV-S^Alpha^ and rVSV-S^Beta^. The IC50 values against rVSV-S^Alpha^ and rVSV-S^Beta^ were comparable but slightly lower than rVSV-S^WT^, suggesting that these mutation combinations may confer reduced susceptibility to serum neutralization (Figure 5E). We also assessed the activities of eight mAbs targeting RBD against rVSV-S^Alpha^ and rVSV-S^Beta^. For neutralization rVSV-S^Alpha^, the activity of mAb-111, mAb 121-C, and mAb 769 was markedly impaired (~10-fold) (Figure 5F). For neutralization rVSV-S^Beta^, the activities of six antibodies were significantly decreased. These results revealed that rVSV-S^Beta^ was more resistant to RBD-specific neutralizing antibodies.

**Figure 6:**
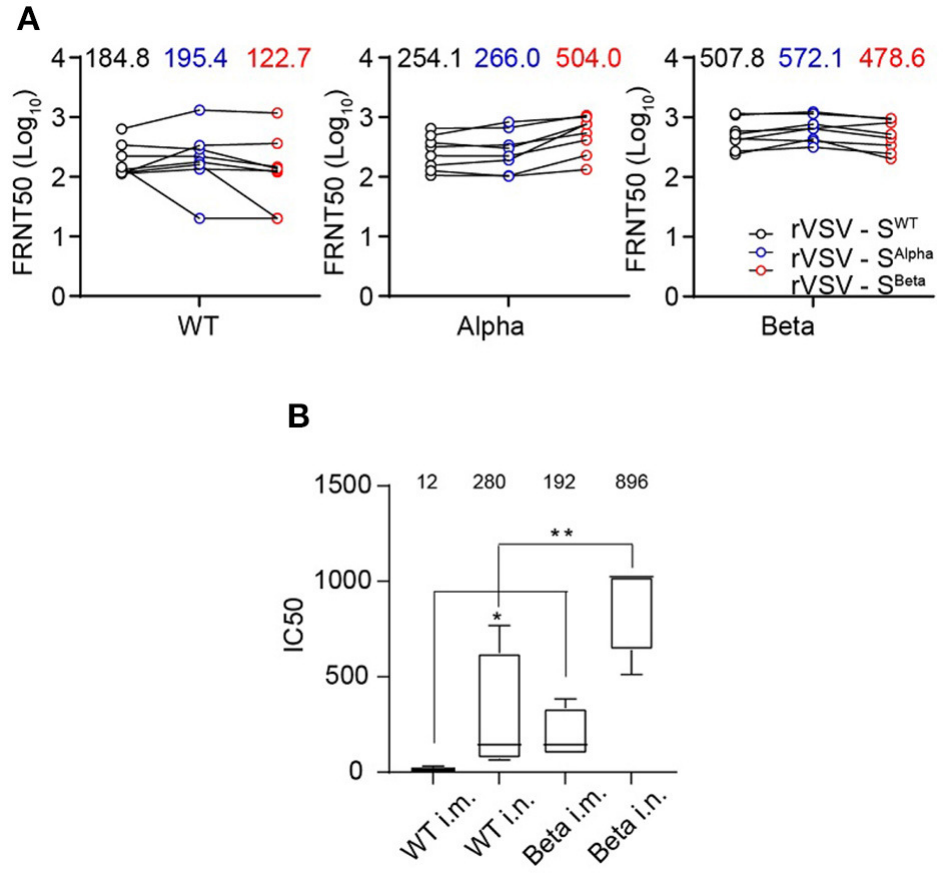
Immunogenicity of recently emerged variants of concern, Alpha and Beta. **(A)** Immunogenicity of SARS-CoV-2 S Alpha and Beta variants. Groups of female *hACE2* knock-in ICR mice (*n* = 8) were vaccinated with a single dose of rVSV-S^WT^ or variants *via* i.p. route (1 × 10^6^ FFU/animal). NAb titers against rVSV-S^WT^, Alpha, and Beta variants were determined by FRNT and calculated by the Reed-Muench method at 30 days post-vaccination. Group GMTs were indicated. All the results are representative of three independent experiments. **(B)** 10^5^ PFU rVSV-S^WT^ and rVSV-S^Beta^ variant were immunized into golden Syrian hamster separately by intramuscular and intranasal injection (*n* = 5). Sera were collected at 30 days post-immunization and detected for neutralizing capacity by CPE reduction against authentic WT SARS-CoV-2 (IC50). Group GMTs were marked in the graph. Statistical significance was evaluated by unpaired two-tailed student's *t* test. **p < 0.01, *p < 0.05. Error bars indicate standard deviation of the mean. All neutralization assays were performed in triplicate.

We further vaccinated hACE2 knock-in mice with rVSV-S^WT^, rVSV-S^Alpha^, and rVSV-S^Beta^ to evaluate their immunogenicity. Mice serum samples were collected 28 days after a single i.p. injection of 1 × 10^6^ FFU of rVSVs (Figure 6A). Each serum was measured for neutralization against rVSV-S^WT^, rVSV-S^Alpha^, and rVSV-S^Beta^. Sera from the rVSV-S^WT^ vaccinated group showed similar neutralizing activity against rVSV-S^WT^ and rVSV-S^Alpha^, but lost approximately 50% activity against rVSV-S^Beta^. Sera from the rVSV-S^Alpha^ vaccinated group was similarly effective in neutralizing rVSV-S^WT^ and rVSV-S^Alpha^. However, it became about two-fold more effective in neutralizing rVSV-S^Beta^. Sera from the rVSV-S^Beta^ vaccinated group showed comparable neutralizing titers against the three viruses. Notably, rVSV-S^Beta^ elicited the most potent neutralizing antibody responses in mouse models, followed by rVSV-S^Alpha^ and then rVSV-S^WT^. The immunogenicity of rVSV-S^Beta^ was further tested in golden Syrian hamsters via intramuscular and intranasal route as described previously (39). rVSV-S^Beta^ elicited significantly higher neutralizing titers than rVSV-S^WT^ against the authentic SARS-CoV-2 WT strain by both routes (Figure 6B). These results indicated that the SARS-CoV-2 S protein-carrying mutations could be improved immunogens to use in the next-generation design of vaccines.

## Discussion

During the COVID-19 pandemic around the globe, many SARS-CoV-2 variants harboring S protein mutations have emerged and raised great concerns about their potential to evade immune responses. Here, we systematically evaluated the antigenicity and immunogenicity of eight S variants using a replication-competent recombinant VSV system. The S variants include five single-mutation variants that emerged with high frequency early in the pandemic, one dominant mutation D614G (40) and two variants of concern, Alpha and Beta, with D614G included (41, 42). Although the neutralizing activity of some mAbs was substantially altered against several variants, all the variants maintained neutralization sensitivity to polyclonal serum samples from patients who recovered from natural infection. However, as the pandemic continues to spread, the frequency of the five mutations we studied (V367F, G476S, V483A, D839Y, and D936Y) has significantly decreased from May 2020 onwards, suggesting that they failed to adapt to the host environment. In contrast, D614G, which exhibited enhanced infectivity (40, 43), has become the dominant form since March 2020 (40, 44, 45). In addition, the two variants of concern, Alpha and Beta, containing novel mutations in addition to D614G emerged, showing altered immunogenicity and potential of escape from current vaccines.

D614G is the first dominant mutation and is correlated with high viral loads in the patients (40). In addition, the D614G strain was more infectious, as revealed by the pseudotyped SARS-CoV-2 system (40). In keeping with the previous results, we also found that the titer of rVSV-S^D614G^ was 9-fold higher than that of the wild-type.

There are some contradictory results about the binding affinity of ACE2 with SARS-CoV-2 variants using different technologies. Via a cell-based soluble ACE2 inhibitory assay, we found the binding affinity between soluble ACE2 and Alpha or Beta was increased by 1.8- or 4.7-fold as compared with WT. In keeping with our results, Ramanathan et al. applied a protein-based microscale thermophoresis assay and found that Alpha variant bound ACE2 with 1.98-times greater affinity than WT, while Beta bound ACE2 with 4.62-times greater affinity, supporting the evidence of higher affinity to ACE2 in Beta variant (46). However, using surface plasmon resonance, Barton et al. analyzed the effect of all single mutation on ACE2 and RBD binding and they found N501Y and E484K mutations enhanced the affinity, while mutation K417N would decrease the affinity of RBD/ACE2 interaction, on the contrary. Their results reveals that ACE2 bound Alpha stronger than Beta variant (47).

The accumulation of mutations in the S protein affect its antigenicity, resulting in the escape from vaccines or therapeutic antibodies (17, 48). All the five early mutations, including V367F, G476S, V483A, D839Y, and D936Y, plus the domain mutation D614G, show similar sensitivity to sera from a convalescent patient infected by WT SARS-CoV-2. The neutralizing activity of the RBD mAbs was also affected by mutations outside the RBD, such as D614G, D839Y, and D936Y, probably due to the change of overall structure of the S protein (37, 49, 50). However, the Alpha and Beta variants showed a close to two-fold decrease in the ID50 value. The variants showed a more significant differential reaction to RBD-specific mAbs, while variants Alpha and Beta were resistant to some antibodies. These results suggest that a cocktail of mAbs targeting separated epitopes is necessary to develop therapeutic antibodies against COVID-19.

rVSV-S is a live-virus vaccine candidate (51, 52). Thus, we tested the immunogenicity of selected variants by immunization of the rVSV-S^WT^ and variants in hACE2 mice by the i.p. route. All the early mutations and the D614G showed immunogenicity similar to that of WT, except D839Y. rVSV-S^D839Y^ immunized sera showed 8-fold decrease in neutralizing itself than WT with ambiguous mechanism, although effects on spike cleavage by TMPRSS2 and interaction with human T cells were reported (36, 50). Each serum sample was assayed for rVSV-S^Alpha^ or rVSV-S^Beta^ for neutralization against the rVSV-S^WT^, rVSV-S^Alpha^, or rVSV-S^Beta^. All these S variants were able to induce neutralizing antibody responses as potent as the wild-type S, indicating that the immunogenicity of the variants was not impaired. The sera from the rVSV-S^WT^ immunized group exhibited reduced neutralizing activity against Beta, as other re searchers reported for natural infection and vaccines induced human samples (17, 53, 54).

Usually, immunized sera or plasma from patients neutralize homologous strain better than heterologous strains. However, rVSV-S^Alpha^ immunization yielded higher neutralization against Beta than against Alpha variant. Further, rVSV-S^Beta^ elicited a higher level and more cross-reactive neutralizing antibodies than the other two viruses in both mouse and hamster models. This was consistent with the observation that plasma from Beta infected patients could cross-neutralize both D614G and Beta variant, while some samples showing three-times higher potency against Gamma than original Beta variant (55). Similar results were observed in vaccines using Beta variant. A booster dose of a Beta strain Moderna vaccine candidate (mRNA-1273.351) yields higher titers against Beta and gamma. ChAdOx1-vectored Beta vaccine (AZD2816) also showed cross-reactive immunogenicity against Beta, delta, and kappa (56). However, a recent preprint reported that Beta variant infected patients revealed reduced cross-neutralization of non-VOC variants (57). Overall, these results highlighted the potential of S^Beta^ as an immunogen for the second generation of COVID-19 vaccines.

## Data Availability Statement

The original contributions presented in the study are included in the article/Supplementary Material, further inquiries can be directed to the corresponding author.

## Ethics Statement

The studies involving human participants were reviewed and approved by State Key Laboratory of Pathogen and Biosecurity, Institute of Microbiology and Epidemiology, Academy of Military Medical Sciences. The patients/participants provided their written informed consent to participate in this study. The animal study was reviewed and approved by Bioethics Committee of the Institute of Zoology, Chinese Academy of Sciences.

## Author Contributions

AZ and YoZ designed the study. AZ supervised the whole project. LD, YuZ, DW, JM, HY, HL, SD, and FY performed the experiments. AZ and FY wrote the manuscript. All authors contributed to the article and approved the submitted version.

## Funding

This project was funded by the Key Research Program of the Chinese Academy of Sciences (KJZD-SW-L06-02), the State Key Research Development Program of China (2019YFC1200500), and the COVID-19 Research Program from the Institute of Zoology, CAS (E0517011).

## Conflict of Interest

The authors declare that the research was conducted in the absence of any commercial or financial relationships that could be construed as a potential conflict of interest.

## Publisher's Note

All claims expressed in this article are solely those of the authors and do not necessarily represent those of their affiliated organizations, or those of the publisher, the editors and the reviewers. Any product that may be evaluated in this article, or claim that may be made by its manufacturer, is not guaranteed or endorsed by the publisher.
