# Supplementary material for: Growth, Antigenicity, and Immunogenicity of SARS-CoV-2 Spike Variants Revealed by a Live rVSV-SARS-CoV-2 Virus
Source: Front Med (Lausanne). 2022 Jan 7;8:793437. doi: 10.3389/fmed.2021.793437 (PMC8777026; doi:10.3389/fmed.2021.793437)
Supplement: Supplementary file 1 [file Presentation_1.pdf]

Supplementary Material

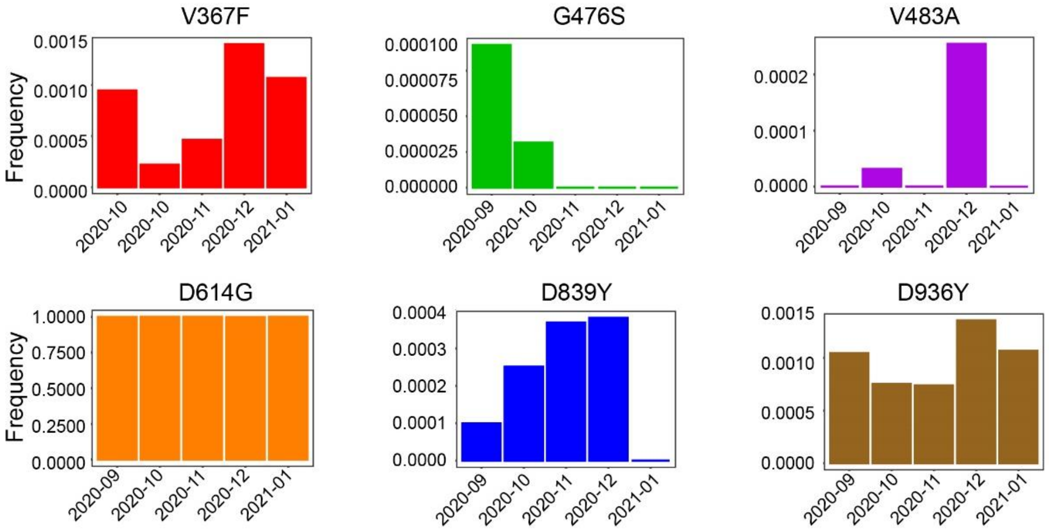

Supplementary Figure 1. Frequencies of SARS-CoV-2 mutations from September 2020 to January 2021, as demonstrated in the histogram.

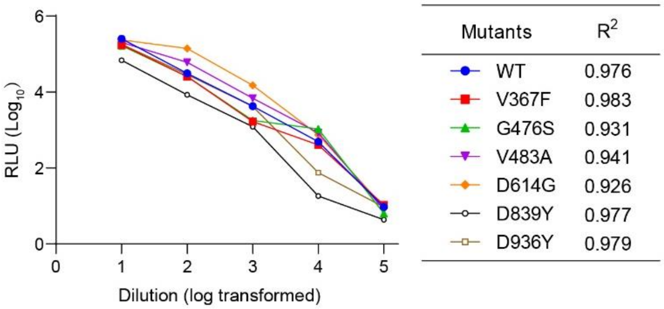

Supplementary Figure 2. Correlation of virus dilution and luciferase value. Vero cells were infected with seven variants at a 10-fold dilution from 10 to 100,000 in triplicate. Relative luminescence units (RLUs) at each dilution were normalized to log-transformed formation. Correlation coefficient numbers were analyzed by GraphPad Prism 9.0 software. The graph is representative of three independent experiments.

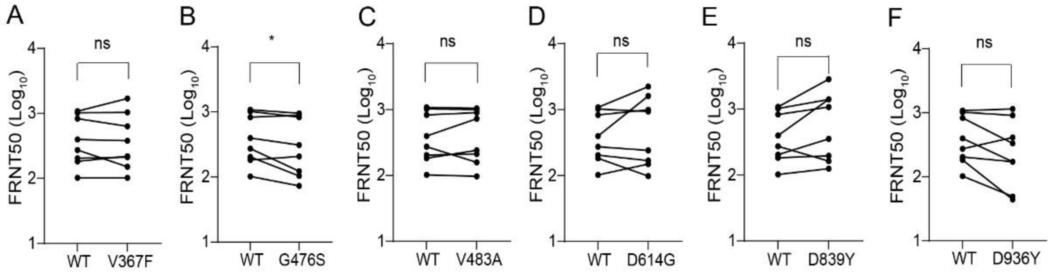

Supplementary Figure 3. Neutralizing activity of eight serum samples from convalescence patients against rVSV-S^{WT} and variants. Significances were analyzed by GraphPad Prism 9.0 software. *P < 0.05.

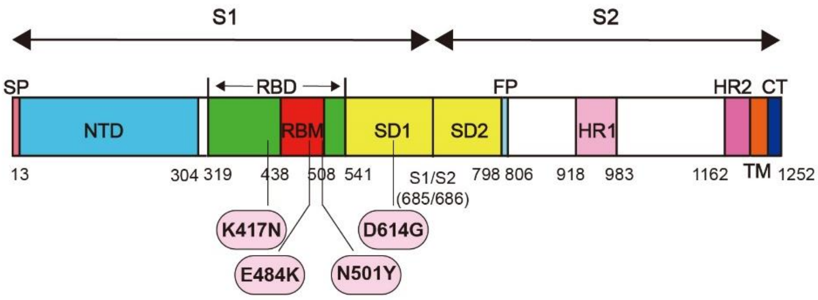

Supplementary Figure 4. (A) Schematic diagram of the SARS-CoV-2 S protein. The mutations in rVSV-S^{Alpha} (N501Y and D614G) and rVSV-S^{Beta} (K417N, E484K, N501Y, and D614G) are marked.
